# A Myopathy, Lactic Acidosis, Sideroblastic Anemia (MLASA) Case Due to a Novel PUS1 Mutation

**DOI:** 10.4274/tjh.2017.0231

**Published:** 2017-12-01

**Authors:** Çiğdem Seher Kasapkara, Leyla Tümer, Nadia Zanetti, Fatih Ezgü, Eleonora Lamantea, Massimo Zeviani

**Affiliations:** 1 Gazi University Faculty of Medicine, Division of Metabolism, Ankara, Turkey; 2 Fondazione IRCCS Carlo Besta, Molecular Neurogenetics Unit, Milan, Italy; 3 Medical Research Council, Mitochondrial Biology Unit, Cambridge, United Kingdom

**Keywords:** Myopathy, Lactic acidosis, Sideroblastic anemia

## To The Editor,

The patient, the first child of Turkish first-cousins, was born at term after an uncomplicated pregnancy. Birth parameters were normal. The family history was negative for hematological or neurological diseases. The newborn period was characterized by hypoglycemia, lactic acidemia (6.1 mmol/L; normal values: up to 1.9 mmol/L), and lactic, pyruvic, and dicarboxylic aciduria. At 10 months of age, hematological examination revealed marked sideroblastic anemia. He started to receive transfusions every 3-4 weeks until 14 months of age, when the blood parameters spontaneously normalized. He had exercise intolerance and delayed motor milestones (walking at 3.5 years of age). At 14 years of age, pallor, progressive muscle weakness, and lethargy occurred and sideroblastic anemia reappeared. The boy had mild mental insufficiency, profound generalized hypotrophy and weakness, and hyperlordosis of the trunk. He became transfusion-dependent, requiring packed cell transfusions every 2-3 weeks. The muscle biopsy showed subsarcolemmal abnormal mitochondrial aggregates and diffuse negative staining for cytochrome c oxidase. Due to the paucity of tissue, the biochemical evaluation of respiratory chain complexes was not performed. The clinical features oriented us towards a mitochondrial pathology; CoQ10 was given (400 mg/day) and dramatic improvement of muscle strength was observed with reduction of the frequency of blood transfusions. Unfortunately, the boy died when he was 18 years old due to severe respiratory failure.

Myopathy, lactic acidosis, and sideroblastic anemia (MLASA) is a rare mitochondrial disease [1]: MLASA1 (MIM #600462) results from mutations in the PUS1 gene, encoding for pseudouridylate synthase 1, and the enzyme is located in both the nucleus and mitochondria, which is involved in post-transcriptional modification of cytoplasmic and mitochondrial tRNAs [2]; MLASA2 (MIM #613561) is caused by mutations in the YARS2 gene that encodes for the mitochondrial tyrosyl-tRNA synthetase [3]; and MLASA3 (MIM #500011) is caused by heteroplasmic mutation m.8969G>A in the mitochondrial-DNA-encoded ATP6 gene (MTATP6) [4].

**Figure 1:**
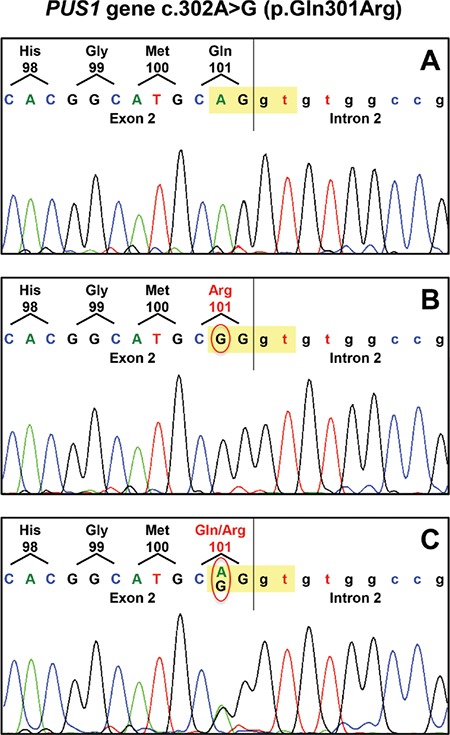
Sequence analysis of the exon 2-intron 2 junction of the PUS1 gene in a control (A), in the patient (B), and in the patient’s mother (C). The yellow highlighted nucleotides belong to the consensus sequence of the splice donor site. In the red circle the mutated nucleotide is in homozygous (B) or heterozygous (C) form.

Our patient had typical features of MLASA, so we analyzed the nuclear-encoded genes YARS2 and PUS1. YARS2 was normal, but we identified the novel homozygous mutation c.302A>G in exon 2 of the PUS1 gene, causing the substitution p.Gln101Arg in the protein (Figure 1). This variant is reported as a singleton in the ExAC browser, accounting for 0.001% of allele frequency, and absent in dbSNP and EVS databases. The p.Gln101Arg change was scored very high for the likelihood to be deleterious by different bioinformatics tools for predicting pathogenic variants, and furthermore the c.302A>G transition is predicted to modify the consensus sequence of the splice donor site of exon 2, probably affecting splicing (Table 1). The unavailability of the patient’s cells did not allow us to confirm this hypothesis.

To date, eleven PUS1-mutated patients from six families have been described [2,5,6,7,8] and five mutations are reported. Ours is the first alteration allegedly causing a splicing aberration according to prediction by in silico analysis. In our patient a high dose of CoQ10 improved the clinical condition for a while, although it did not reverse the course of the disease. To date, there is no effective therapy for MLASA, although many studies are in progress to address novel treatment options for mitochondrial diseases [9]. Our report expands the genetic spectrum of the MLASA syndrome, which must be considered in patients with congenital sideroblastic anemia associated with myopathy.

## Figures and Tables

**Table 1:**
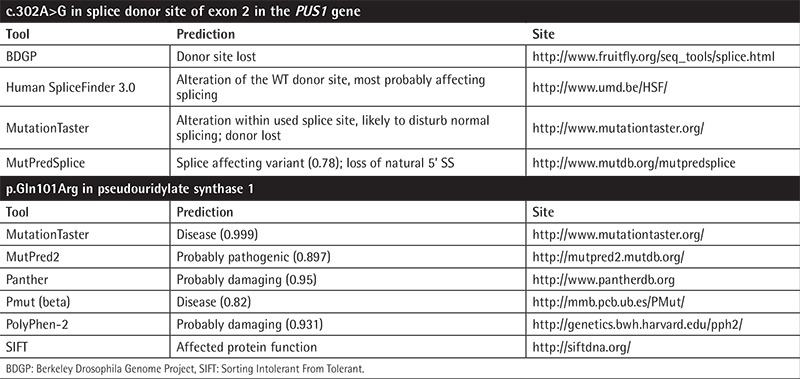
Results of the in silico analysis obtained by different bioinformatics tools for the prediction of the impact of mutation on mRNA and protein.
